# Developing and Testing a Middle-Range Theory of the Well-Being Supportive Physical Environment of Home-Dwelling Elderly

**DOI:** 10.1155/2013/945635

**Published:** 2013-05-13

**Authors:** Satu Elo, Maria Kääriäinen, Arja Isola, Helvi Kyngäs

**Affiliations:** Institute of Health Sciences, University of Oulu, P.O. Box 5000, 90014 Oulu, Finland

## Abstract

The aim is to describe the development of a middle-range theory by using an inductive-deductive approach. A theory of well-being supporting physical environment of home-dwelling elderly is used as an example. The inductive-deductive theory development process is described through four different phases: (1) the creations of concepts were described inductively through concept synthesis, (2) relationships between the concepts were examined to set up a hypothetical model, (3) hypotheses were set up to verify the concepts and to test hypothetical models, and (4) the verification and presentation of the theory.

## 1. Introduction

Nursing science theories can be divided into metatheories, conceptual models, and middle-range and small theories [1, 2]. Each level of theory has its own characteristics and aims to separate it from the rest [3]. The need for middle-range theories arose in the 1960s in the field of sociology. In nursing science, they have been used to narrow the gap between nursing science theories and practice [4, 5]. Particularly in the 1990s, the development of middle-range theories became relatively common on an international level [5, 6]. Middle-range theories may be explanatory or predictive [7, 8] and they are more limited in their nature than metatheories or conceptual models. They also contain fewer concepts and are situated between micro/-practical theories and conceptual models [8–10]. A middle-range theory may be developed using either a deductive or an inductive approach [11] and, unlike metatheories or conceptual models, it can be operationalized into a measurable form [12].

Nursing science theories can be developed using either an inductive or a deductive approach. When developing a theory, these stages often occur in turns, and the development process may begin using either an inductive or a deductive approach, depending on the nature of previous research data. An inductive approach to developing a theory is used when the aim is to form a theory on a subject on which little information is available, if the information is fragmentary or if the aim is to find a new viewpoint on the subject. When forming a theory using inductive methods, individual observations are used as the basis. Based on several individual observations, conclusions may be drawn and a theory constructed [2, 13]. When forming a theory using deductive methods, general conformity rules are used as starting point, divided into smaller units, and rendered into concrete form. Therefore, the deduction process progresses from general to individual level [13]. The methods of quantitative study are widely used in deductive theory formation. From the viewpoint of forming a theory, it is significant that the researcher has a theoretical presumption concerning the subject on which the theory is being formed. The presumption is based on previous information. In developing a theory, both inductive and deductive approaches are often used. This is called inductive-deductive or deductive-inductive theory formation, depending on whether the starting point of the theory formation is inductive or deductive [2, 12].

Theories can be classified based on their type or level [2]. When theories are considered according to type, they can be divided into descriptive, explanatory, predictive, and directive [15]. Descriptive theories describe, observe, and name concepts but do not explain the relationships between them. Therefore, descriptive theories do not investigate the effect that a change in one concept has on another concept within a theory [16]. Instead, after identifying and naming the phenomenon, an explanatory theory looks into the causal relationships between concepts. They are used in an attempt to obtain answers to the question of to what extent the phenomenon occurs simultaneously or together with another or how the two are connected [2]. The aim of explanatory theories is to explain how or why concepts are connected through cause and effect relationships as well as various correlations. Predictive theories describe in detail the associations between concepts with the aid of direct or indirect cause and effect relationships [1]. They are used to estimate possible outcomes of different situations. The aim of directive theory, in turn, is to present alternative solution and action models in order to achieve the best possible end results [14].

Developing nursing science theory through research has become increasingly more common since the 1980s. However, studies on the subject were not published in scientific journals until the 1990s. Around that time, discussion arose on the significance of different-level theories in the advancement of science [17]. Through theory development, facts and information can be organised in a systematic manner. Theory defines the nature, structure, concepts, and associations between concepts of a real-life phenomenon. Nursing science theories may be described as a group of concepts, definitions, and statements on the topic of nursing-related phenomena [7]. They can be used for critical observation of phenomena related to nursing and to develop evidence-based nursing [18].

**Figure 1:**
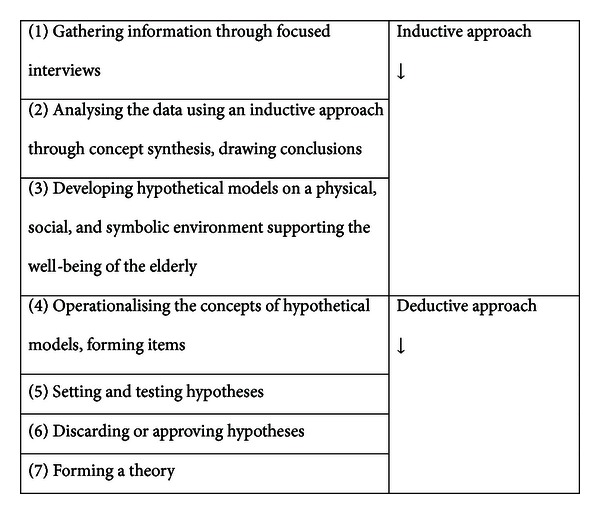
The phases of forming a theory, adapted from Lauri & Kyngäs 2005 [14].

The purpose of this paper is to describe the development of a middle-range theory by using an inductive-deductive approach. The example theory has been developed according to the following phases: (1) selection of concepts and their synthesis, (2) definition of relationships between concepts, (3) setting and testing hypotheses, and (4) presentation and verification of the theory. The first two phases represent an inductive approach and the last two a deductive approach. The phases are presented in Figure 1. The development of the theory is illustrated with an example. The example used here is a theory on a well-being supportive physical environment of home-dwelling elderly people [19, 20]. In the study in question, a descriptive and explanatory middle-range theory was formed based on the experiences of the elderly.

## 2. The Process of Developing an Inductive-Deductive Theory

### 2.1. A Theory of the Well-Being Supportive Physical Environment

The study used as an example looks at well-being from the viewpoint of self-perceived or subjective well-being, meaning that the well-being of the elderly is evaluated by the elderly themselves. Following the principle of inductive theory formation, well-being was not defined in advance: the definition was based on the experiences of the study subjects. The starting point was that the environment was considered as a source of well-being, with the elderly seen as fulfilling their needs and the environment as a resource that contributes to well-being. 

The main concepts of the well-being supportive physical environment include an environment that enables safe activity, a pleasant physical environment, and Northern environment. An environment that enables safe activity comprises both safety at home and immediate surroundings that enable safe mobility. At home, important aspects include the safety of stairs and steps, reducing the need to reach or climb, the use of various support rails, and making floors and the bathroom less slippery. In the immediate surroundings of the home, ensuring safe mobility calls for well-maintained traffic routes and the use of technical aids. A pleasant physical environment consists of tidiness at home and in its immediate surroundings, closeness to natural environment, and opportunities for various activities. Natural environment areas, such as parks and gardens, are popular meeting places that provide an opportunity to interact with other people. They are also popular among the elderly for exercise and relaxation. A pleasant living environment also includes possibilities to engage in various activities. Several factors associated with Northern environment, such as climate and availability of services, are related to the well-being of the elderly. For example, various cold- or heat-related symptoms during the winter or summer make everyday coping more difficult and weaken the perceived state of health of the elderly [19, 20].

### 2.2. An Inductive Phase

Formulation of concepts was the first phase of theory development and concepts were formed through concept synthesis. The aim of this was to observe the concept in a systematic and disciplined manner by an inductive approach [2]. An inductive approach was selected, because the key concepts (well-being and environment) are abstract. In addition, there is little previous research information available on an environment promoting well-being in the context of ageing, and the definition of the concept of environment has largely been based on findings by researchers and theoreticians in fields other than nursing science [11].

 The concept synthesis is based on a qualitative approach, with interview data as a basis. The interview data were gathered from home-dwelling elderly people over the age of 65 in Northern Finland (*N* = 39). The interviews were carried out as focused interviews, with loose themes prepared in advance directing the course of the interview. The interviews proceeded with descriptions of dwelling history and current living environment used as introductory topics. This was followed by the interviewees describing in their own words the elements of a physical environment that promotes well-being. If necessary, the interviewer asked for specifications with focused themes, based on the classification of environment in Kim's (2000) typology [15].

After transcribing the interview data, concept synthesis was used to construct concepts based on the information gathered from individual observations by grouping and organising information regarding the phenomenon [21]. The aim was to answer the question of what are the attributes of a physical environment that support well-being.

Through concept synthesis, the aim was to construct models representing the phenomenon under study in a condensed form by producing concepts that describe the phenomenon. The analysis was begun by selecting a unit of analysis—a letter, a word, a sentence, or a longer semantic unit, depending on the aims of the study at hand [21]. In the present study, the unit of analysis chosen was a semantic unit describing experiences of well-being related to environment. When reading through the data, questions were asked according to the aims of the study: what are the attributes of physical environment that support well-being? At the same time, notes were made in the margins as answers to the questions were found. The material was read through several times until no more notes were made in order to describe all the attributes of the concepts [22]. After this, the original statements and the notes in the margins concerning them were classified into categories according to different subareas of the environment, and the grouping of synonymous expressions within each subarea was commenced.

**Figure 2:**
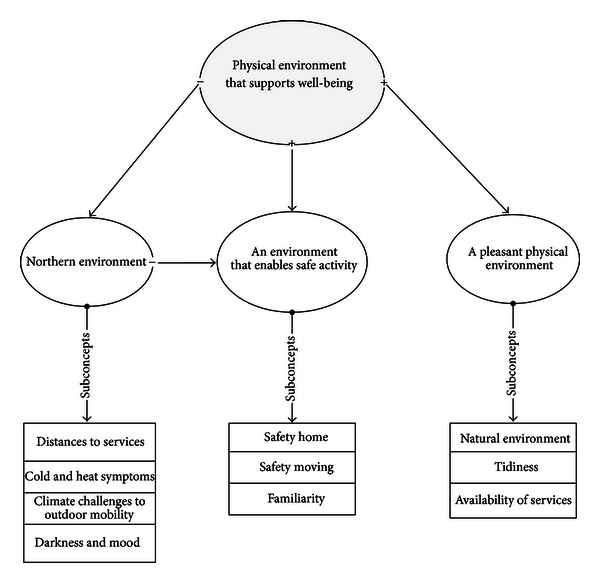
An example of a hypothetical model.

In order to form concepts, synonymous statements were merged to form subconcepts, and these were given names that described them well [21]. The subconcepts were further merged to form concepts, and the concepts merged to form main concepts and possibly connective concepts as well. When forming concepts using an inductive method, the researcher interprets the data and, based on the interpretation, decides which points can or cannot be merged to form sub- or concepts or main concepts [9]. As a result, concepts with related contents for hypothetical model were achieved according to the principles of inductive theory formation. The hypothetical model on the well-being supportive physical environment of home-dwelling elderly people was formed. The relationships between concepts were described as a hierarchy of concepts comprising all the attributes of an environment that supports well-being. An example of the hypothetical model is shown in Figure 2.

### 2.3. A Deductive Phase

In a deductive phase the hypotheses for testing the theory are set and tested [14]. The hypothesis set for physical environment was as follows: the structures of the main concepts relating to the physical environment: (1) Northern environment, (2) pleasant physical environment, and (3) environment that enables safe activity can be indicated.

In order for hypotheses to be tested, there must be an instrument that can be used to test the concepts presented as hypotheses [2, 14]. In such cases, the concepts of the hypothetical models were operationalized. After a concept was defined, its relationships with other concepts occurring in the theory were investigated by forming statements. The total amount of 100 statements connected the concepts to each other [2, 8]. The statements were placed on a five-point scale.

The reliability of a theory calls for a valid instrument. Pretesting plays a significant part in assessing the validity of the indicator [23]. The instrument was pretested using expert evaluation (one expert on methodology and two experts on contents), panel evaluation (15 nursing science students in the final stages of their studies), and postal questionnaire data (*n* = 96). The panel and expert evaluations were used to identify the items in the instrument that were misunderstood or poorly worded, and these were improved with further instructions [23]. Both were used to determine whether the concepts used in the instrument complied with the theory with regard to content and whether they were comprehensive enough in describing the phenomenon in question [24]. The panel of experts made use of the unanimity coefficient content validity indexes (CVI). An instrument was considered internally consistent or homogeneous because the panel's unanimity rate was >80% [25]. After the panel and expert evaluations, the revised instrument was sent to home-dwelling elderly people for pretesting. The purpose of the analysis of the pretesting data gathered through a postal questionnaire was to look at the correlation coefficients between items in order to identify any poorly formulated items. All correlations were sufficiently high (*r* > 0.30), and no items were omitted due to low correlations. After pretesting, the instrument set up to test the theory consisted of 100 items. 

To test the theory, a pretested version of the instrument was sent in first phase to 500 home-dwelling elderly people between ages 65 and 74 in Northern Finland. The stratified random sampling method was used. Based on the data (*n* = 328), the model constructed using an inductive method was tested using principal component analysis as well as confirmatory factor analysis. Exploratory factor analysis (EFA) is suited for the initial stages of the study, when the researcher is still uncertain as to how many factors need to be explained in the model [26]. The purpose of principal component analysis was to verify the subconcepts constructed using an inductive method within each subarea of environment. The aim was to condense the variables, that is, items, of the indicator into a few factors or principal components with content relevance [23]. Principal component refers to a group of intercorrelating variables that were subconcepts of the theory. 

The principal component analysis was carried out in four phases. First, the correlation coefficients and covariances between items that were high enough to allow analysis (>0.30) were calculated. In the second phase, estimated factor loadings of the principal components/factors were obtained for the matrix formed based on the correlation coefficients and covariances. Items with loadings over 0.40 were included in the factors. In the literature, recommendations for factor loadings to be included in the analysis vary between 0.35 and 0.55 [23]. In the third phase, the factor loadings were rotated in order to facilitate their interpretation. Rotation facilitates interpretation, as its aim is to load each item on only one factor. The analysis was performed using rectangular varimax rotation, meaning that the factors were independent from each other.

After the analysis, the goodness of the principal components was estimated in terms of content and based on the factor loadings of the items. The goodness of individual items loaded on the principal components was investigated using communalities. Communality reveals how strongly the item is loaded on the principal component, giving an item with strong loading a communality value close to 1. Items not fulfilling the requirements of goodness may be removed based on low communalities. The main principle is that the communality of an item should be >0.30. The communalities of all the items exceeded this value, indicating that they measure the factors in a relatively reliable manner. All the factors were relevant in terms of content, though some differed from the hypothetical model. For example, factors related to living comfort of the natural environment and tidy environment were loaded on the same factor. All the Northern environment factors that impair mobility were also loaded on the same factor. These alterations were made in the hypothetical model for the purposes of confirmatory factor analysis.

Confirmatory factor analysis (CFA) was used to ensure that the data supported the models constructed through principal component analysis [27, 28]. As in the study used as an example, exploratory factor analysis is usually followed by confirmatory factor analysis when forming a theory. Confirmatory factor analysis is extremely well suited for measuring associations between latent variables, that is, concepts occurring in a theory [29]. CFA enables modification with the aid of indices of relevance to the data [23]. As performing confirmatory factor analysis requires an identifying model, one item in each factor was given the value 1 and the others 0. The values of the model's parameters could be estimated using the method of maximum credibility. After this, the models were ready to be tested. The associations between concepts as well as the model's suitability to the data were observed with the aid of indices. CFA is based on correlation and covariance matrices. The starting point of analysis is that items that are related to each other should have higher correlation coefficients than items that are not thought to be related [23].

After CFA, all factors, that is, principal components, were named. They can be named according to the item or items with the highest loading and according to the processes contributing to the origin of the factors. However, from a theory-developing point of view a good alternative is to base the naming on theory. In this study, naming was based on the results of the first phase of the study, during which theoretical concepts were formed. The theoretical concepts describing the physical environment as well as their alpha values are presented in Table 1.

Developing a theory is a process that should not end with presenting the theory; the theory should be tested at different time points and with different target groups. Based on confirmatory factor analysis results the theory of the well-being supportive physical environment has been verified in three different data during years 2005–2012 (Table 2). The theory presented in this paper was also developed further by complementing it from two different points of view [30]. The theory was tested and developed further from two different points of view in order to add depth to the theory. With the first data set (data 1), the theory was tested among respondents over the age of 75 years (*n* = 539) in order to allow comparison of results between two different age groups. With another data set (data 2), the theory was tested among elderly people living in Southern Finland (aged 65–74 years, *n* = 372), which allows a wider perspective when looking at the Northern dimension of the environment. The theory was tested by confirmatory factor analysis (CFA) and results of indexes of the goodness of fit were presented. Based on all data the main concepts of the well-being supportive physical environment (Northern environment, an environment that enables safe activity, and a pleasant physical environment) were scientifically verified. There were no notable differences between a place of residence or age groups. The results show that the theory can be generalized across the older home dwelling elderly and the elderly living in Southern part of Finland.

## 3. Discussion

### 3.1. Applying the Theory in Practice

A widely used criterion in evaluating a theory is applying the theory in practice [18]. A theory developed in a manner typical of middle-range theories may be used in nursing education and as a tool for thinking by nursing staff. The concepts can be utilised in analysing and defining an environment that supports the well-being of the elderly. The consequences that can be derived from the theory are related to directing research and practical work, producing new ideas and separating the key focus of interest of nursing from that of other fields [31]. The theory of an environment supporting the well-being of elderly people is a middle-range theory that can be applied to other studies. In such cases, the concepts of the theory guide further studies of different kind. For example, further studies may look at how much the different environments in which the elderly live actually contribute to their well-being. After this, the theory can be used as a guideline in practical nursing work or for giving various recommendations to develop it.

The theory was tested with confirmatory factor analysis which showed that the validity and reliability of the theory was relatively good. The statistical values as well as the indices of relevance to the data presented as a result of the analysis are sufficient in all the models tested. Using factor analysis in testing the theory was found to be a successful solution, and the indices obtained in the analysis confirmed the hypothetical models formed by the researcher through qualitative analysis.

### 3.2. The Reliability of the Theory

The theory was tested with confirmatory factor analysis which showed that the validity and reliability of the theory were relatively good. The statistical values as well as the indices of relevance to the data presented as a result of the analysis are sufficient in all the models tested. Using factor analysis in testing the theory was found to be a successful solution, and the indices obtained in the analysis confirmed the hypothetical models formed by the researcher through qualitative analysis. Cronbach alpha values are shown in Table 1.

## 4. Conclusions

This paper also gives an example of how to develop and verify an inductive-deductive nursing science theory. Theory development is multiphases process. Here the theory is based on inductive approach which provides perspective of elderly people. After inductive phase there was a need to test and verify the theory by deductive approach. Advanced statistical methods enable us to study the relationships between concepts and build the theories. Middle-range nursing theories are needed to develop the practice and nursing science itself.

## Figures and Tables

**Table 1:** Names, alpha values, and number of items of the concepts describing physical environment (*n* = 328).

Concept	Cronbach's alpha	Number of items
Alterations in the home facilitating domestic activities	.94	3
Natural environment contributing to living comfort	.82	3
Factors of Northern environment complicating mobility	.84	3
Temperature as a factor complicating life	.68	2
Natural environment as a pleasant location for exercise	.80	3
Darkness as a factor promoting depression	.93	2
Well-maintained traffic routes facilitating mobility	.67	2
Availability of services as a prerequisite for living at home	.65	2
Meeting familiar people in a natural environment	.60	2
Moving in the immediate vicinity	.50	2
A tidy environment contributing to living comfortably	.62	2

**Table 2:** The results of confirmatory factor analysis (data 1–data 3) as indices of relevance to the data.

Model and data	Indices of relevance								
	*χ* ^2^	df	*χ* ^2^-*p*-value	GFI	AGFI	RMR	NFI	CFI	RMSEA
Northern environment, four-factor model									
Data 1: Northern Finland 75–85	49	21	<.00	.98	.96	.05	.97	.98	.05
Data 2: Southern Finland 65–74	40	19	<.00	.98	.95	.06	.97	.98	.05
Data 3: Northern Finland 65–74	340	21	.01	.98	.95	.05	.97	.99	.05
An environment that enables safe activity									
Data 1: Northern Finland 75–85	8	11	.68	.99	.99	.3	.99	1	<.00
Data 2: Southern Finland 65–74	10	11	.57	.99	.98	.02	.99	1	<.00
Data 3: Northern Finland 65–74	7	11	.80	.99	.99	.04	.99	1	<.00
A pleasant physical environment									
Data 1: Northern Finland 75–85	60	28	<.00	.98	.96	.04	.96	.98	.05
Data 2: Southern Finland 65–74	75	29	<.00	.96	.93	.06	.94	.96	.07
Data 3: Northern Finland 65–74	52	30	.008	.97	.95	.02	.95	.98	.05
